# Effect of Processing Parameters on the Heating Uniformity of Postharvest Tobacco Leaves Subjected to Radio Frequency Disinfestations

**DOI:** 10.3390/insects16020228

**Published:** 2025-02-19

**Authors:** Jinsong Zhang, Yingqi Tian, Xin Ye, Zijun Mo, Rui Li, Shaojin Wang

**Affiliations:** 1 College of Mechanical and Electronic Engineering, Northwest A&F University, Yangling 712100, China; zhangjinsong@nwafu.edu.cn (J.Z.); tianyingqi@nwafu.edu.cn (Y.T.); yexin@nwafu.edu.cn (X.Y.); mozj@nwafu.edu.cn (Z.M.); ruili1216@nwafu.edu.cn (R.L.); 2 Department of Biological Systems Engineering, Washington State University, Pullman, WA 99164-6120, USA

**Keywords:** postharvest pest control, heating rate and uniformity, radio frequency heating, tobacco leaves, product quality

## Abstract

Uneven heating is the biggest obstacle to the application of radio frequency (RF) technology in insecticidal applications. Since many existing studies have focused on the RF heating uniformity of granular materials, few RF treatment solutions have been reported for leaf materials. To improve the RF heating uniformity of tobacco leaves, a combined RF-hot air treatment method was developed for disinfestation in this study. The experiments were conducted to determine the effects of three process parameters (electrode gap, sample thickness, and hot air temperature) on the RF heating rate and uniformity of tobacco leaves. The results showed that the heating rate of RF-hot air combined treatment increased with decreasing electrode gap but increased with increasing sample thickness and hot air temperature. The RF heating uniformity in tobacco leaves decreased with increasing heating rate but could be significantly improved with the conveyor movement. Considering the heating uniformity and heating rate, the material thickness of 60 mm and electrode gap of 110 mm were selected as optimal process parameters. The results of the study may provide a feasible method to improve the RF heating uniformity in tobacco leaves and potential applications for effective disinfestations.

## Simple Summary

Physical heating methods to kill pests are currently given high attention due to avoiding the environmental pollution caused by chemical methods. Radio frequency (RF) heating, as an efficient and rapid dielectric heating method, has been demonstrated in many studies to successfully control pests, such as rice weevils and grain beetles, in a variety of major grain-based stored products. However, most of the existing reports lack RF insecticidal studies on leafy materials, and it is essential to explore the appropriate RF heating parameters and analyze their effects on the heating rate and heating uniformity before proceeding with the validation of insecticidal efficacy. The results showed that better heating uniformity and faster heating rate could be obtained under the conditions of hot air temperature of 55 °C and conveyor speed of 11.8 m/h. These conditions can be used as process parameters for RF disinfesting tobacco leaves.

## 1. Introduction

China is an important tobacco growing and producing country in the world. According to the statistics of the Food and Agriculture Organization (FAO) of the United Nations, China’s tobacco leaves production has shown a slow growth trend in recent years but has ranked first in the world for many years. China’s tobacco leaves production reached 2180 kilotons in 2022, accounting for about 36% of the world totals (FAOSTAT, 2022). Currently, in China, harvested tobacco leaves are stored in constant temperature and humidity warehouses for one to two years for fermentation. The great infestation risk of insect pests during long storage may cause huge losses and quality degradations of tobacco leaves [1]. Chemical fumigation has been widely used in the world for disinfesting agricultural products, including tobacco leaves, due to its simple operation and low cost. Concerns about harmful chemical residues, increased resistance, and environmental pollution have forced the tobacco industry to seek physical methods to replace chemical fumigation [2,3].

In recent years, many advanced thermal processing technologies have been proposed as alternative physical methods, thus avoiding the possible hazards of chemical methods. Radio frequency (RF) treatment is a promising method for disinfesting postharvest agricultural products due to its rapid and volumetric heating [4,5,6]. The principle is that the high-frequency alternating electric field makes the ions and polar molecules in the electric field rotate and move, thus generating heat by friction. The principle of this disinfestation method is to heat the material and pests at the same time. Because the pests have a higher moisture content, the temperature of pests would be higher than that of host material after RF heating for the same time, so as to kill pests at a lower material temperature. This emerging technology has been widely used for controlling insect pests in beans [7,8], grains [9,10,11], nuts [4,12,13], and dry fruits [14,15]. Especially for industrial applications, the 27.12 MHz, 25 kW RF systems have been successfully used to control insects in in-shell walnuts with acceptable product quality and high throughput [16,17]. These studies have demonstrated that the RF heating technology could be a potential physical method for disinfesting tobacco leaves with acceptable product quality. 

There are two major pests in tobacco leaves: *Lasioderma serricorne* (F.) (Coleoptera: Anobiidae) and *Ephestia elutella* (Hübner) (Lepidoptera: Pyralidae). The main damage stage of these two pests is the larvae, causing huge economic losses by eating tobacco leaves or polluting tobacco leaves with excreta [18]. There are still non-negligible problems about the non-uniform heating in the RF treatment of agricultural products, such as overheating in the corners of rectangular products [19,20] and uneven distribution of the electromagnetic field, leading to insufficient cold spots to control all pests and reduce the product quality at the hot spot [21,22,23]. These heating non-uniformity problems may hinder the large-scale application of RF technology in the field of agricultural products processing. Heating uniformity is mainly affected by the physical properties of the sample itself and the processing conditions, such as geometry shape [24,25], hot air assistance [26,27], conveyor movement [28,29], and sample rotation [30,31]. Before conducting the efficacy experiments, the RF heating uniformity needs to be determined and improved under the optimized processing conditions, such as thicknesses for tobacco leaves, electrode gaps, and auxiliary conditions. 

The objectives of this study were to: (1) determine effective RF process parameters, such as electrode gap, sample thickness and heating time for efficient treatment of tobacco leaves, (2) evaluate the RF heating rate and uniformity of tobacco leaves under different process parameters, (3) compare the effect of RF heating uniformity of tobacco leaves under different auxiliary conditions (hot air at different temperatures and conveyor belt movement), and (4) evaluate the energy efficiency of the given RF treatment.

## 2. Materials and Methods

### 2.1. Tobacco Leaves Preparation

The tobacco leaves used in the experiment were provided by Qujing Redrying Factory in Yunnan, China. After 12 h of humidification, these tobacco leaves were manually classified into three grades (high, medium, and low) according to their quality. The samples used in this experiment were all of a medium quality. The initial moisture content (MC) of tobacco leaves was determined by moisture meter (MC-T, Brabender Co., Ltd., Shanghai, China), and tobacco leaves with an MC of 14.65% ± 0.36% on wet basis (w.b.) were selected as test samples. The sample leaves in the length direction were cut to fit into a rectangular container (590 mm × 390 mm × 60 mm), and the cut samples were placed evenly into the container for the subsequent RF treatments.

### 2.2. RF Heating System

**Figure 1:**
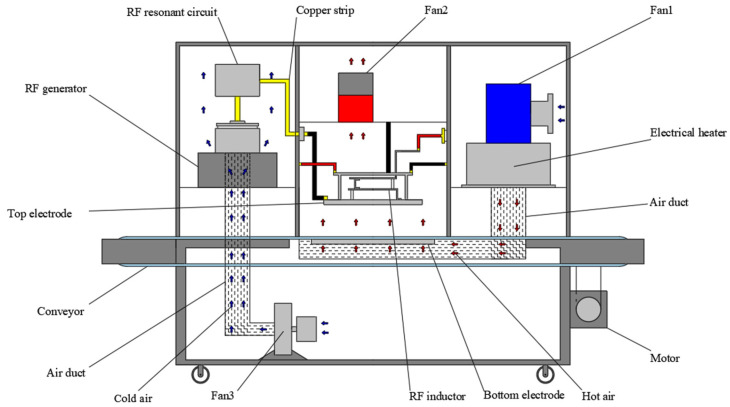
Schematic diagram of hot air-assisted radio frequency heating equipment.

A 10 kW, 27.12 MHz pilot-scale free-running oscillator RF system (10RFS, Hagong Jinlang Co., Ltd., Hefei, China), together with a customized auxiliary hot air system (3 kW), was used for heating tobacco leaves (Figure 1). The size of the parallel perforated electrode plates was 600 mm × 450 mm. The RF output power can be regulated by moving the top plate to adjust the electrode gap from 100 mm to 200 mm. A conveyor belt between electrodes was used to simulate continuous processes for moving samples during RF heating. The speed of the conveyor belt can be set from 0 to 120 m/h to meet the required residence times of different products. The assisted hot air system blew air upwards from the bottom electrode, and the hot air temperature range was from room temperature (25 °C) to 55 °C.

### 2.3. Determination of Electrode Gap and Conveyor Belt Speed

**Figure 2:**
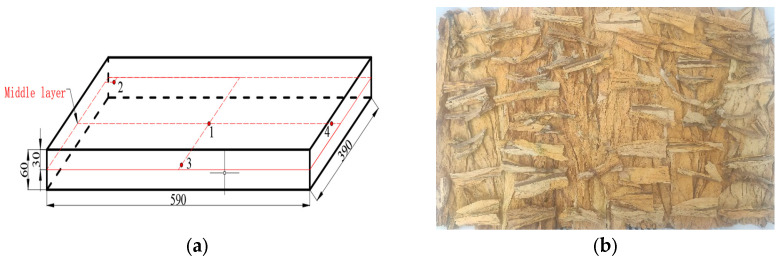
Schematic diagram of the sample container (all dimensions are in mm) (**a**), and the real picture for filling tobacco leaves in a rectangular container (**b**).

**Figure 3:**
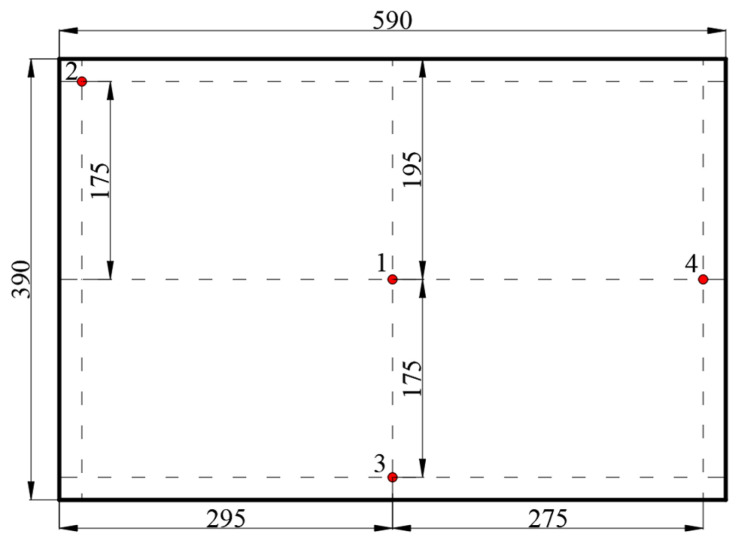
Locations of four fiber optic sensors (1–4) in the middle layer of the samples (all dimensions are in mm).

To develop a continuous RF treatment protocol, tobacco leaves with thicknesses of 40, 60, and 80 mm were placed in three plastic containers on the conveyor belt above the bottom electrode. A layer of tobacco leaves was laid equidistant from each other at the bottom of the container, and a further layer of tobacco leaves was laid above the gap between the two leaves of the first layer, thereby forming a second layer of tobacco leaves. After filling two layers of tobacco leaves, a layer of cut stems was placed evenly on top of the two layers until the set sample thickness was achieved (Figure 2b). To determine the influence of electrode gaps on the RF heating rate, the anodic current (*I_a_*, A) that is linearly related to the RF power was observed under various electrode gaps from 100 to 140 mm with an increment of 5 mm but without the conveyor belt movement and hot air heating. The control group was set up as empty containers without tobacco leaves and placed in the same position. After switching on the RF device, the electrical current on the ammeter of the RF system was immediately recorded under three sample thicknesses, and then the RF unit was turned off. Based on the measured anodic current, three suitable electrode gaps (105, 110, and 115 mm) were selected based on the required heating rate for further temperature–time history experiments. The corresponding temperature profiles at the four locations in the container (Figure 2a) were recorded from ambient temperature (25 °C) to 55 °C under three sample thicknesses. The final temperature was selected based on the target one for controlling most insects in agricultural products [4,11]. Temperature changes in the sample during RF heating were measured by four fiber optic temperature sensors (HQ-FTS-D120, Heqi Technologies Inc., Xi’an, China) with an accuracy of ±0.5 °C. The temperature measurement points were located in the middle layer of the plastic container, and the specific location distribution is shown in Figure 3. The final electrode gap was determined according to the target heating rate (about 5 °C/min) of the tobacco leaves. Three replications were performed for each set of trials. The conveyor belt speed during the continuous RF heating was calculated by dividing the electrode length by the heating time.

### 2.4. Evaluation of RF Heating Uniformity

The RF heating uniformity in tobacco leaves is crucial to developing an effective treatment protocol. The averages and standard deviations of temperatures on the top sample surface were used to evaluate the RF heating uniformity, which has been successfully applied in corn [30], jujube [32] and other agricultural products [10,33]. The heating uniformity index (*λ*) could be calculated using the following equation [34]:

(1) $$\lambda =\frac{\sqrt{\sigma^{2}-\sigma_{0}^{2}}}{\mu -\mu_{0}}$$

where *μ* and *μ*_0_ are the final and initial average sample temperatures (°C), *σ* and *σ*_0_ are the final and initial standard deviations (°C) of the sample temperatures over treatment time, respectively. The smaller *λ* values represent the better uniformity of the RF heating.

The RF heating uniformity index was first determined under three material thicknesses (40, 60, and 80 mm) and three electrode gaps (105, 110, and 115 mm) without hot air and conveyor movement. Then, the uniformity index was evaluated for exploring the effect of three hot air temperatures (45, 50, and 55 °C) with the determined electrode gap of 110 mm and the sample thicknesses of 60 mm under stationary conditions. The target temperature of 55 °C was selected based on 100% mortality of tobacco insects achieved at 48, 28, and 8 min at 51, 53, and 55 °C, respectively [35]. The RF heating uniformity index was finally determined under three complementary methods with the same electrode gap and sample thickness, including RF heating alone, RF heating under conveyor movement, and RF heating with conveyor movement and hot air heating at 55 °C. For the conveyor movement during the RF heating process, three containers filled with tobacco leaves were placed on a conveyor belt to simulate the continuous heating process. Since the electric current increased as the first container moved in between the electrodes and stabilized when the container was completely filled under the electrodes and decreased as the third one moved out from between the electrodes, the second container was selected for mapping the surface temperature and calculating the RF heating uniformity index so as to prevent the effects of transient heating conditions. After RF treatments, the temperature at the top of the tobacco leaves was measured using an infrared camera (A300, FLIR System, Inc., North Billerica, MA, USA) with an accuracy of ±2 °C. The temperature data of the samples were used for calculating the RF heating uniformity index.

### 2.5. Analysis of Energy Efficiency for the RF Heating System

The heating efficiency calculations for the RF system were based on the second container. The current was recorded and used to estimate the RF power when the second container was completely located between electrodes. The input power of the RF system was calculated from the anode current (*I_a_*, A) of the system according to the formula provided by the equipment manufacturer:

(2) $$P(I)=5840I_{a}-2600I_{a}^{2}$$

*P(I)* is the input power (W) that varies with the input current and was estimated to be 1518 W. The RF heating efficiency (*η*, %) is the ratio of total output power (*P_output_*, W) to the input one (*P_input_*, W), which could be calculated by the total energy absorbed by the tobacco leaves according to the recorded sample temperatures, and estimated RF power with the energy from the hot air system. The energy efficiency could be determined according to the other research [8,17]:

(3) $$\eta =\frac{P_{output}}{P_{input}}\times 100\%=\frac{mC_{p}(\frac{\Delta T}{\Delta t})}{P(I)+Ah(T_{a}-\bar{T_{s}})}\times 100\%$$

where *m* is total mass (kg) of tobacco leaves, *C_p_* is the average specific heat capacity of the tobacco leaves, and was taken as 2421.3 J/kg °C in this study [36], *ΔT* is the temperature increase (°C) in the tobacco leaves during the treatment time, *Δt* is the RF processing time (s), *A* is the surface area (m^2^) of samples passed by the hot air, *h* is the convective heat transfer coefficient of the hot air, and was taken as 28 W/m^2^ °C [37], *T_a_* is the temperature (°C) of hot air, and was taken as 55 °C, and $\bar{T_{s}}$ is the average temperature (°C) of the sample surface during the RF heating period, and was taken as 38.6 °C.

### 2.6. Statistical Analysis

All results were expressed as the mean with standard deviation of the data over three independent replicates. The means of temperature and heating uniformity index *λ* were evaluated by analysis of variance (ANOVA) using IBM SPSS Statistics 25.0 software, and the significant (*p* ≤ 0.05) difference was determined by the Tukey test.

## 3. Results and Discussion

### 3.1. Anode Current of RF System Under Different Electrode Gaps and Sample Thicknesses

**Figure 4:**
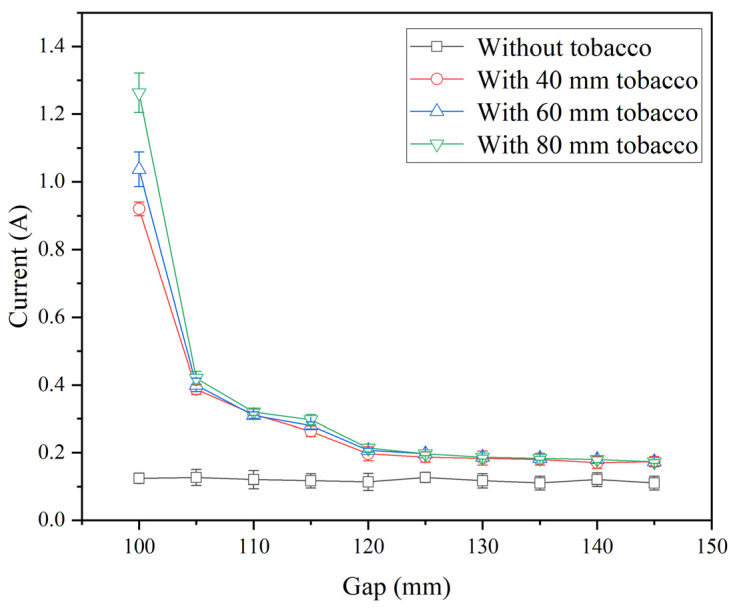
The relationship between the anode current of the RF system and electrode gaps under the material thicknesses of 40, 60, and 80 mm without conveyor movement and hot air heating.

The relationship between anode current and electrode gap is shown in Figure 4 when three containers with or without tobacco leaves were used under three sample thicknesses without conveyor belt movement and hot air heating. The electrical current maintained almost constantly and fluctuated between 0.11 and 0.13 A under different electrode gaps without samples. With samples, however, the electrical current decreased sharply under the small electrode gaps (100–120 mm) and slowly for large gaps (120–145 mm). The maximum electrical current under the smallest electrode gap (100 mm) increased with the increasing sample thickness from 40 to 80 mm (Figure 4). The same trends were also found by [33,38]. To obtain relatively high stability of RF power and suitable heating rate in industrial applications, three electrode gaps (105, 110, and 115 mm) were selected for further RF heating treatment.

### 3.2. Effect of the Electrode Gap and Sample Thickness on the RF Heating Rate and Uniformity

**Figure 5:**
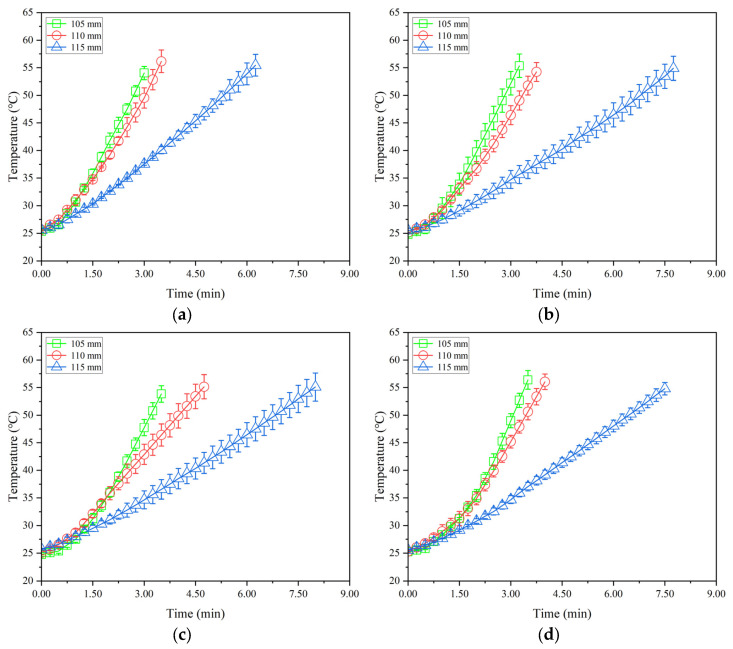
Temperature–time histories of four points ((**a**–**d**) represent the points 1, 2, 3, and 4 in Figure 2, respectively) in tobacco leaves with thicknesses of 40 mm under the electrode gaps of 105, 110, and 115 mm without conveyor movement and hot air heating.

**Figure 6:**
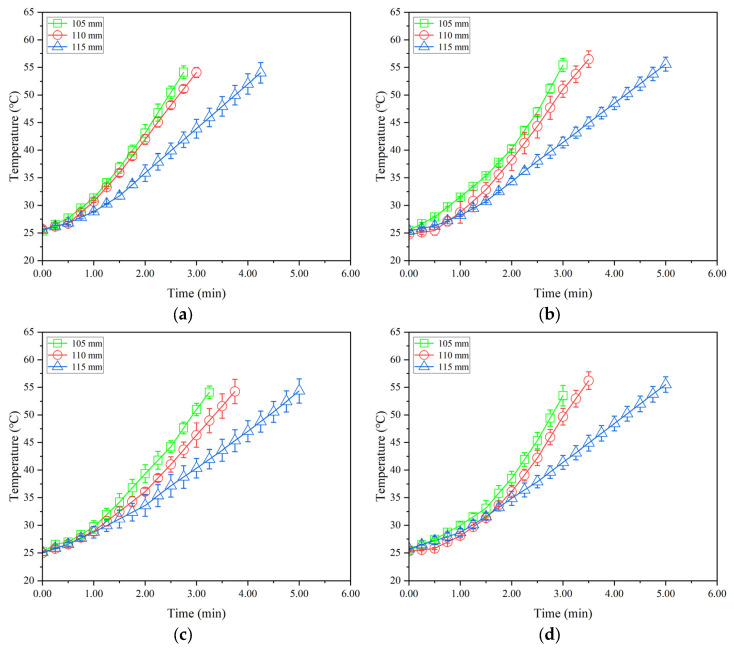
Temperature–time histories of four points ((**a**–**d**) represent the points 1, 2, 3, and 4 in Figure 2, respectively) in tobacco leaves with thicknesses of 60 mm under the electrode gaps of 105, 110, and 115 mm without conveyor movement and hot air heating.

**Figure 7:**
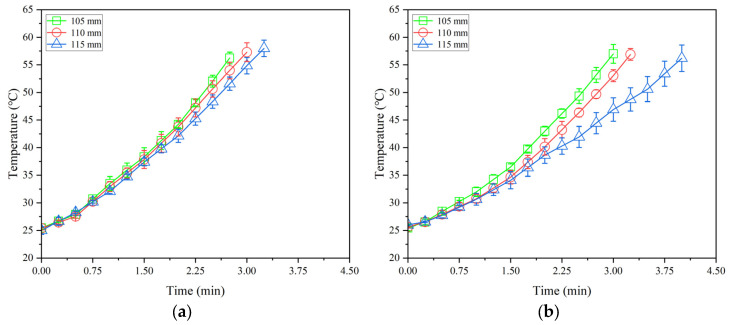

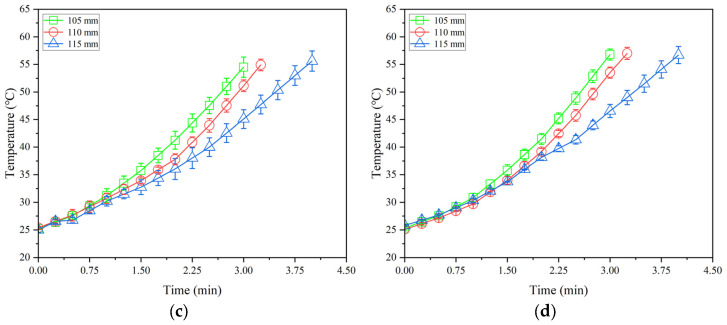
Temperature–time histories of four points ((**a**–**d**) represent the points 1, 2, 3, and 4 in Figure 2, respectively) in tobacco leaves with thicknesses of 80 mm under the electrode gaps of 105, 110, and 115 mm without conveyor movement and hot air heating.

Figure 5, Figure 6 and Figure 7 show the temperature–time histories of tobacco leaves with three thicknesses of 40, 60, and 80 mm in the container during RF heating under three electrode gaps of 105, 110, and 115 mm, respectively. The RF heating rate increased with decreasing electrode gap or increasing sample thickness. About 3.1, 3.4, 3.5, and 3.7 min were needed to raise the sample temperatures from 25 to 55 °C at the given points of 1, 2, 3, and 4, respectively, resulting in the heating rates of 9.7, 8.8, 8.6, and 8.1 °C/min under the electrode gap of 110 mm and the sample thickness of 60 mm (Figure 6a–d). The heating time increased with increasing electrode gap or decreasing thicknesses of tobacco leaves, correspondingly reducing heating rates. The obtained heating rates in this study were higher than those in RF-treated almonds [12] and walnut kernels [39]. Figure 5, Figure 6 and Figure 7 show that the heating rate of the sample at the central point was higher than that at other points. This phenomenon could be attributed to the filling method of tobacco leaves, resulting in more high-temperature stems near the central point. Meanwhile, the existence of air gaps between each layer of tobacco leaves near the internal walls of the container resulted in a greater heat exchange between the tobacco leaves and ambient air.

Table 1 listed a detailed comparison of the heating uniformity index values for three sample thicknesses (40, 60, and 80 mm) and three electrode gaps (105, 110, and 115 mm) after RF heating. Heating uniformity improves with the decreased value of *λ*, and poor heating uniformity means uneven temperature distribution. There was no significant difference in the heating uniformity index when the sample thickness was 40 and 60 mm, but the *λ* value increased sharply when the sample thickness was raised to 80 mm. This was consistent with the trend that the better heating uniformity of samples was obtained under larger electrode gaps [40]. To obtain a relatively high heating rate with acceptable heating uniformity in industrial applications, the electrode gap and sample thickness of 110 and 60 mm were chosen for further RF heating.

### 3.3. Temperature Distributions and Heating Uniformity Index of Tobacco Leaves Under Different Hot Air Temperatures

**Figure 8:**
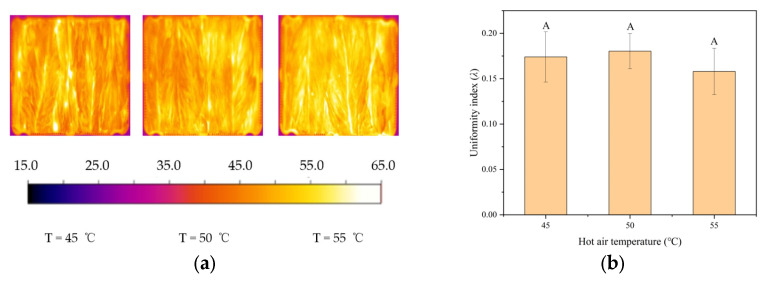
Temperature distributions (**a**) and uniformity index (**b**) of tobacco leaves with thicknesses of 60 mm after RF treatments with electrode gap of 110 mm under different hot air temperatures (45, 50, and 55 °C) and without conveyor belt movement. Same capital letters indicate that the *λ* values are not significantly different (*p* > 0.05) under different hot air temperatures.

Figure 8a shows the temperature distribution of the tobacco leaves under the hot air with three temperatures of 45, 50, and 55 °C. The average temperature of the tobacco leaves increased with increasing hot air temperature, which could be caused by the raised energy gain from the hot air and reduced heat loss to the surrounding air. This similar trend has also been reported in RF-hot air combined drying of paddy [41]. As shown in Figure 8b, hot air heating during RF treatment of tobacco leaves can reduce the value of the RF heating uniformity index, but there was no significant difference (*p* > 0.05) among three hot air temperatures. With the same electrode gap and thicknesses of tobacco leaves, the most obvious improvement in the RF heating uniformity was achieved by 55 °C hot air heating. This trend of reducing the heating uniformity index for RF treatment by hot air heating has also been reported in the combined RF-hot air treatment of hazelnuts [42]. This suggested that the hot air temperature of 55 °C could be applied to develop the effective RF treatment protocol.

### 3.4. Effect of Different RF Treatment Methods on Uniformity

**Figure 9:**
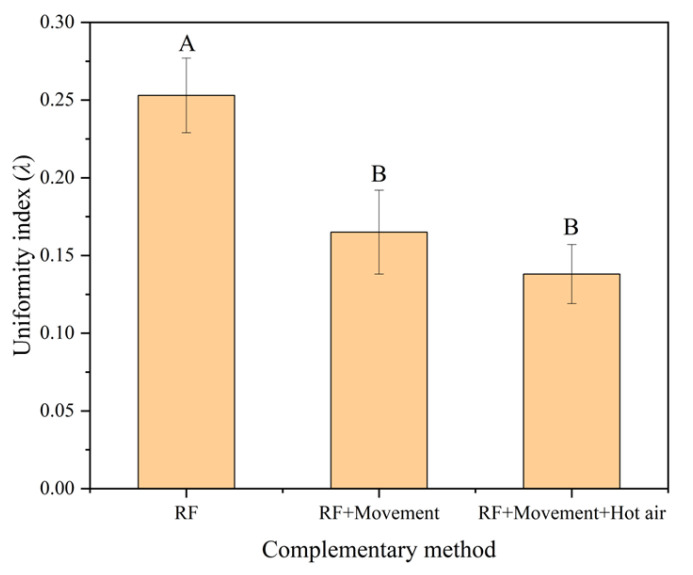
Uniformity index of tobacco leaves with thicknesses of 60 mm under different RF treatment methods under electrode gap of 110 mm and hot air temperature of 55 °C. Different capital letters indicate that the *λ* values are significantly different (*p* < 0.05) under different complementary methods.

Figure 9 demonstrates the comparison of the RF heating uniformity index among three RF treatment methods under an electrode gap of 110 mm, sample thickness of 60 mm, and hot air temperature of 55 °C. The RF heating uniformity index of the tobacco leaves decreased with the added conveyor belt movement and further by the combination of hot air heating and conveyor belt movement. There was a significant (*p* < 0.05) difference in the heating uniformity index between these two methods and RF treatment alone. A similar trend could be found in moved samples reported by other researchers [43,44]. The overall results suggested that RF heating with a combined conveyor movement and hot air treatment was selected to achieve the required temperature distribution for tobacco leaves. The heating time was determined to be 3.1 min, which was calculated by the heating rate (9.7 °C/min) and the temperature difference (30 °C) before and after RF heating. Based on the RF heating time, the average speed of the conveyor belt was, therefore, set to 11.8 m/h.

### 3.5. Evaluation of Thermal Efficiency of RF Heating System

According to Equation (3), the thermal efficiency of the RF system was calculated to be 75.6% after determining the electrode gap, sample thickness, hot air temperature, and conveyor belt speed as 110 mm, 60 mm, 55 °C, and 11.8 m/h, respectively. The thermal efficiency was comparable to that (72.5%) of the RF treatment of milled rice [11] and was higher than that (60.0%) of the laboratory-scale RF treatment for walnuts [13] but lower than that (84.6%) of microwave heating for rice [45]. By comparing the low thermal efficiency (10%) of a hot air dryer for apple slices [46], it can be concluded that the hot air assisted RF treatment may provide a potential disinfestation method for tobacco leaves with the required heating uniformity and energy efficiency.

## 4. Conclusions

In this study, the RF treatment process parameters were determined to meet the actual industrial applications, and their effects on the RF heating uniformity and energy efficiency were also evaluated. The results showed that the heating rate of tobacco leaves increased with the decrease in electrode gap or the increase in sample thickness. The uniformity of RF heating for tobacco leaves decreased with increasing heating rate and was improved by either conveyor belt movement or hot air heating. The high energy efficiency (75.6%) and acceptable heating uniformity provide a reliable basis for industrial applications. The future study would focus on raising the throughput of RF treatment and developing an effective RF protocol based on insect mortality and product quality.

In this study, the electrode gap (110 mm), sample thickness (60 mm), hot air temperature (55 °C), and conveyor belt speed (11.8 m/h) were determined as suitable RF treatment process parameters, and their effects on the RF heating uniformity and energy efficiency were also evaluated. The results showed that the heating rate of tobacco leaves increased with the decrease in the electrode gap or the increase in sample thickness. The uniformity of RF heating for tobacco leaves decreased with increasing heating rate and was improved by either conveyor belt movement or hot air heating. The high energy efficiency (75.6%) and acceptable heating uniformity provide a reliable basis for industrial applications. The future study would focus on raising the throughput of RF treatment and developing an effective RF protocol based on insect mortality and product quality.

## Figures and Tables

**Table 1:** Uniformity index values of tobacco leaves under different electrode gaps and sample thicknesses without hot air heating convey belt movement.

Gap (mm)	Thickness of Tobacco Leaves (mm)		
	40	60	80
115	0.236 ± 0.015 Ba *	0.251 ± 0.011 ABa	0.274 ± 0.019 Aa
110	0.239 ± 0.020 Aa	0.253 ± 0.024 Aa	0.302 ± 0.025 Aa
105	0.243 ± 0.021 Ba	0.256 ± 0.027 Ba	0.364 ± 0.030 Ab

* Different lower- and uppercase letters indicate that means are significantly different (*p* < 0.05) under different electrode gaps and sample thickness, respectively.

## Data Availability

Data will be made available on request.
